# Myocardial infarction and alcohol consumption: A case-control study

**DOI:** 10.1371/journal.pone.0198129

**Published:** 2018-06-04

**Authors:** Milena Ilic, Sandra Grujicic Sipetic, Branko Ristic, Irena Ilic

**Affiliations:** 1 Department of Epidemiology, Faculty of Medical Sciences, University of Kragujevac, Kragujevac, Serbia; 2 Institute of Epidemiology, Faculty of Medicine, University of Belgrade, Belgrade, Serbia; 3 Clinic for Orthopedic Surgery and Traumatology, Clinical Center Kragujevac, Faculty of Medical Sciences, University of Kragujevac, Kragujevac, Serbia; 4 Faculty of Medicine, University of Belgrade, Belgrade, Serbia; Universidad Miguel Hernandez de Elche, SPAIN

## Abstract

**Background:**

Although epidemiological evidence for the beneficial effect of low alcohol consumption on myocardial infarction is strong, the impact of heavy drinking episodes is less clear.

**Objectives:**

The aim of this study was to investigate a possible association between the risk for acute myocardial infarction occurrence and alcohol consumption.

**Methods:**

Our hospital-based case-control study comprised 374 participants (187 newly diagnosed patients with myocardial infarction and 187 controls, individually matched by gender, age, and place of residence). This study was performed in Kragujevac (a city in Serbia) during 2010. Logistic regression analysis was used to determine odds ratio (OR) with 95% confidence intervals (95% CI).

**Results:**

The history of alcohol consumption in patients with acute myocardial infarction and their controls did not differ significantly: the percentage of those that were consuming alcohol was slightly higher in cases (54.5%) than in controls (50.3%). The habit of binge drinking during the previous 12 months was significantly more common in cases (25.1%) than in controls (12.8%): adjusted OR = 2.2 (95%CI = 1.2–4.2, *p* = 0.017), *p* for trend = 0.015. Analysis of binge drinking by age, gender and place of residence revealed that the increase in risk for acute myocardial infarction was associated with older age (adjusted OR = 5.1, 95%CI = 1.7–15.1, *p* for trend = 0.010), male gender (adjusted OR = 2.3, 95%CI = 1.1–5.2, *p* for trend = 0.028) and rural place of residence (adjusted OR = 4.8, 95%CI = 1.3–18.5, *p* for trend = 0.033).

**Conclusion:**

Our results suggest that binge drinking is associated with twice the risk for myocardial infarction compared to not drinking. Since consumption of alcohol is very common in the Serbian population, the effect of binge drinking on myocardial infarction should be considered an important public health issue.

## Introduction

Myocardial infarction represents death of myocard cells due to irreversible ischemia progressing to necrosis [1,2]. According to the World Health Organization’s estimates, every year approximately 6 million people around the world experience a myocardial infarction, and the lethal outcome occurs in over 25% of cases [1]. Mortality rates for myocardial infarction are dropping in North America and many North and West European countries, while in Central and East Europe these rates are increasing [3]. In 2014, over 16560 people (10270 men and 6290 women) in Serbia suffered a heart attack, and over 5000 people died (around 3000 men and 2000 women) [4,5]. It is estimated that around two thirds of the myocardial infarction mortality rates decline in developed countries are due to reduced exposure to risk factors, while the last third is the result of adequate treatment and improved survival [6].

Etiology of myocardial infarction is complex and still not completely elucidated [7,8]. Age, smoking, hypertension, high cholesterol levels, diabetes mellitus and male sex are recognized as major risk factors for myocardial infarction [8]. Other risk factors include obesity, physical inactivity, dietary factors, positive family history and psychosocial factors. However, in some developing countries, the contribution of established myocardial infarction risk factors is not entirely known. The results available to date suggest a significant presence of conventional risk factors for acute myocardial infarction not only in patients, but also among controls (especially those under 60 years of age) [9–11]. The “INTERHEART” study, which involved participants from 52 countries, pointed out that in different parts of the world different risk factors were responsible for the development of myocardial infarction: factors which showed the strongest link with the development of myocardial infarction in Africa were diabetes and hypertension, in South America it was obesity and smoking, while the most important risk factors in Croatia were current smoking, diabetes, higher ApoB/Apo A-1 ratio, obesity and hypertension, while alcohol consumption was found to be protective [12].

Numerous epidemiological studies showed that moderate alcohol consumption might decrease cardiovascular mortality [13, 14] and was associated with reduced cardiovascular risk [15–17]. Tavani et al [18] found that alcohol consumption inversely correlated with non-fatal myocardial infarction, independent of the type of alcohol beverage, but related to the duration of alcohol consumption. A population-based case control study in Spain showed that moderate alcohol consumption (up to 30 mg/day: including wine, beer and spirits) was related with a reduction in risk for non-fatal myocardial infarction, while heavy alcohol intake did not significantly reduce the risk [15]. A large study in China that included 64597 persons showed that alcohol consumption led to a lower risk for heart attack [19]. However, the protective effect of moderate alcohol consumption on myocardial infarction occurrence is lost when it is characterized by consumption patterns that include episodes of intake of large quantities of alcohol [10, 20–22].

Although epidemiological evidence for the beneficial effects of low alcohol consumption on myocardial infarction is strong, the impact of heavy drinking episodes is less clear [12,23]. Some studies have shown that heavy drinking, as well as binge drinking, can lead to acute myocardial infarction [23–25]. The “INTERHEART” study showed that episodes of binge drinking can lead to the onset of myocardial infarction and that especially in older people [26]. In one center in Northern Ireland (Belfast) and three centers in France (Lille, Strasbourg, and Toulouse), the hazard ratio for „hard coronary events”(incident myocardial infarction and coronary death) for binge drinkers compared with regular drinkers was 1.97 (95%CI = 1.21 to 3.22) in middle aged men [21]. Risk of a major coronary event for men who consumed ≥ 9 drinks in 24 hours before the onset of symptoms was 2.26 (95% CI = 1.06 to 4.81) compared with regular drinkers who consumed no alcohol in that period [27]. Population-based case–control study in New York [28] showed that frequency of episodic alcoholic intoxication of at least once/month or more was associated with a strongly increased risk of non-fatal myocardial infarction in women aged 35–69 years compared to abstention (OR = 2.90; 95%CI = 1.01–8.29), while compared to current drinkers who never drink to this extent the risk was 6.22 (95%CI = 2.07–18.69). A nested case–control study in Sweden recorded that taking large amounts of alcohol on a single occasion was associated with an increased risk for myocardial infarction in women but not in men [29]. The beer binge drinking is associated with increased risk of fatal acute myocardial infarction in men (Kuopio, Finland) whose usual intake of beer was 6 or more bottles per session compared with men who usually consumed less than 3 bottles (relative risk was 6.50, 95%CI = 2.05–20.61) [30]. Any heavy drinking occasion was associated with a two times higher mortality from ischaemic heart disease in former drinkers compared with former drinkers without such heavy drinking occasions, and in current drinkers compared with current drinkers with average daily intake of one to two drinks [31].

A very small number of analytical epidemiological studies on the etiology of acute myocardial infarction have been conducted in Serbia. The aim of this paper was to assess the link between alcohol consumption and risk for acute myocardial infarction in our population.

## Methods

### Study design

This hospital-based case-control study was performed in Kragujevac (a city in Serbia with about 200,000 inhabitants) during 2010. The Clinical Center in Kragujevac, one of the four clinical centers in the country, provides tertiary health care.

The healthcare system in Serbia is free to access, universal, and covers all citizens and permanent residents. This system is free of direct charge, because all employees must pay contributions to it. The network of health care institutions throughout Serbia is organized on three levels—the primary, secondary and tertiary healthcare providing level. With political and social changes since 2000, there have been some improvements of the health system and general living circumstances in Serbia [32]. The Human Development Index values had a rising trend since 2000 (0.726), reaching 0.767 in 2010. Diagnosis and treatment of ischemic heart disease, especially myocardial infarction, have significantly improved in the last 5–10 years, and Serbia now has an epidemiological pattern similar to that in most of the neighboring countries.

### Study population

The study population consisted of newly diagnosed patients with their first myocardial infarction according to the diagnostic criteria based on the European Society of Cardiology/American College of Cardiology consensus guidelines: specific symptoms of acute myocardial infarction, changes in the blood levels of specific enzymes and/or acute myocardial infarction-specific electrocardiogram changes [2]. All cases were hospitalized at the Clinical Center in Kragujevac. No one refused to participate in the study.

Controls were selected among patients who were hospitalized at the same time in the Clinical Center in Kragujevac, without either a diagnosis or a history of myocardial infarction, or any other cardiovascular disease. Controls consisted of patients who were, due to other diseases or conditions (such as closed or open fracture of the arms, legs or ribs, gonarthrosis, coxarthrosis, etc), hospitalized at the Clinic for Orthopedic Surgery and Traumatology, Clinical Center in Kragujevac. All selected controls were interviewed; no one refused to participate.

Cases and controls were individually matched by gender, age (± 2 years) and place of residence (rural / urban). All cases and controls fulfilled the same criteria for inclusion of participants in the study (they gave voluntary consent and had no criteria for exclusion). Exclusion criteria for the study were age of 18 years or less, positive history of a psychiatric illness, pregnancy and lactation, refusal to participate in research or any other objective reason that did not allow or hindered participation in the study.

### Ethics considerations

This study was conducted according to the guidelines laid down in the Declaration of Helsinki and all procedures involving human subjects were approved by Ethics Committee of the Faculty of Medical Sciences, University of Kragujevac (Ref. No.: 01–529), and by the Ethics Committee of the Clinical Center Kragujevac (Ref. No.: 01–13815). Informed verbal consent was obtained from both cases and controls prior to the interview. Prior to obtaining a verbal consent, the medical doctors who conducted the interview thoroughly informed the participants of the details and purpose of the study, after which the medical doctors conducting the interview signed the questionnaire. At the time when our study was started, the Ethics Committee of the Clinical Center in Kragujevac did not require a written and signed informed consent for studies without any clinical examination that involve only interviews, such as our case-control study. The interviews were always conducted in the hospital. The interview took approximately 2 hours. A questionnaire was completed through face-to-face interviews conducted by trained interviewers (medical doctors). The mean time interval between diagnosis and interview of cases and controls was 2 weeks.

### Sample size calculation

The overall sample calculation was based on the results of previously published studies of similar design. According to the data from the "INTERHEART study", the probability of exposure to the investigated risk factor (alcohol) in the control group was 47.14% [12]. With a two sided probability of the type I error alpha of 5%, based on the power of 80%, the assumed coefficient of correlation for exposure to the examined factor between the matched cases and the controls of r = 0.2, based on the applied matching and cases:controls ratio of 1:1, it is estimated that the minimum sample size required to detect the true odds ratio greater than 2.0 or less than 0.55 is 167 participants in each group. The StatsDirect statistical software (Version 3.0.184, Stats Direct Ltd, 9 Bonville Chase, Altrincham, Cheshire WA14 4QA, United Kingdom) was used to calculate the sample size.

### Data collection

The epidemiological questionnaire and medical records were used as data sources in this study. The data on each participant were collected by a face-to-face interview, which lasted approximately 1.5–2 hours and was conducted by trained interviewers (medical doctors). Besides the questions about socio-demographic characteristics, the questionnaire contained questions on the exposure to potential risk factors and protective factors for acute myocardial infarction, as well as the habit of alcohol consumption (alcohol consumption anytime during lifetime, those who consumed were then asked about frequency, time of the beginning and duration of alcohol consumption, types of alcoholic beverages, average amount of beverages per day, binge drinking, cessation of consumption, duration of abstinence; additionally, we asked about frequency and amount of alcohol intake for 11 types of alcoholic beverages during the last 10 years in the special part of the questionnaire which contained questions of food intake).

Demographic variables included occupation, education level, marital status. Occupation was categorized as manual worker, farmer, clerk, professional and housewife (for retired the occupation before retirement was shown). Education level was categorized as low (≤8 years), and high (>8 years). Marital status was dichotomized as with partner versus without partner. Anthropometric measurements (weight and height) of the participants were taken using the standard instruments and techniques. Degree of obesity was estimated based on the body mass index (BMI): subjects with BMI ≥ 25kg/m^2^ were considered overweight [33]. Data on family history of myocardial infarction, personal medical history (hypertension, diabetes mellitus, hypercholesterolemia, disorders of thyroidea, etc), oral contraceptive use, the presence of stressful events (death, stress in the family, financial stress, stress at work, etc) in the previous 12 months, use of tobacco, coffee and alcohol were collected. Participants were considered as smokers if they regularly smoked at least one cigarette per day or approximately 30 g of tobacco per month for one year. Participants were classified as current smokers if they had smoked at least one cigarette every day for the last 12 months, and as former smokers if at least one year passed since smoking cessation.

Data on alcohol consumption were related to the regular intake of any amounts of these beverages. Participants were also questioned about the frequency of alcohol consumption anytime during lifetime (never, sometimes, every day), quantity of alcohol (standard bottle of beer, as well as a glass of wine and a shot of spirits were used as measures of consumption), age at the time of the beginning of consumption (< 20 years, ≥ 20 years), duration of consumption (< 30 years, ≥ 30 years), cessation of consumption, the duration of abstinence (≤ 10 years, > 10 years). Also, participants were questioned about the habit of drinking alcohol during the last 10 years before the onset of the present illness: the frequency of consumption (daily, weekly, monthly) and the amount of consumption (bottle of 500 ml for beer, glass of 200 ml for wine, shot of 50 ml for spiriths) for 11 types of alcoholic beverages (rakija, cognac, brandy, vodka, whiskey, liqueur, vermouth, white wine, rose wine, red wine and beer). Based on the data on the frequency and amount of consumption, the average individual daily intake for each type of alcoholic beverage was determined. If significant changes in the consumption had happened during the observed period, habits before the change were recorded. Participants were classified as daily drinkers if they had drinked at least one drink every day for the last 12 months, and as former drinkers if at least one year passed since drinking cessation.

The total daily amount of consumption for each drink item was calculated and then finally converted into „pure”alcohol using food consumption tables [34]. In Serbia, a „standard”drink is any drink that contains about 13 grams of „pure”alcohol [34]. Daily quantity of alcohol consumption was categorized based on the total amount of pure alcohol per day (g), into two groups (≤ 13, > 13).

Binge drinking was defined as the consumption of 5+ standard drinks for men and 4+ standard drinks for women on one occasion at least once a month during the last year preceding the onset of the myocardial infarction for cases and the current illness for controls.

### Statistical analysis

A descriptive analysis was performed for categorical variables by using absolute and relative frequencies. Differences were analyzed using the univariate logistic regression model between subgroups of age, gender, place of residence, education level, marital status, body mass index, oral contraceptive use, history of diabetes mellitus, hypertension, hypercholesterolemia, disorders of thyroidea, stressful events, family history of myocardial infarction, cigarette smoking and coffee consumption. Additionally, the univariate logistic regression model was used for analysis of characteristics of patients with acute myocardial infarction and their controls, according to alcohol consumption history.

Univariate and multivariate logistic regression models were performed to estimate the odds ratio (OR) with 95% confidence interval (95% CI) in order to assess the relationships between alcohol consumption and myocardial infarction occurrence. Multivariate logistic regression analysis was used to estimate the adjusted odds ratio with the aim of determining the independent risk factors for myocardial infarction associated with a history of alcohol consumption. Adjustment was made for all variables that were related to myocardial infarction in univariate analyses at a p value of <0.10 (body mass index, positive personal medical history for diabetes mellitus, hypertension, hypercholesterolemia, and disorders of thyroidea, family history of myocardial infarction, stressful events, and tobacco use). Model fit was assessed by the Hosmer-Lemeshow test of goodness of fit and Cox and Snell’s and Nagelkerke’s Pseudo R square measures, together with the calculation of the area under the ROC curve. A test for linear trend in risk was based on the logistic regression model. For binge drinking only, we performed subgroup analyses to explore further the effects of age, gender and place of residence, as the variables known from literature data as possible confounders for the association between alcohol consumption and acute myocardial infarction, although these variables were matched in the present study. Statistical significance was considered when p<0.050. All statistical analyses were conducted using the Statistical Package for Social Sciences software (SPSS Inc, version 20.0, Chicago, IL).

## Results

### Participant characteristics

The case group with acute myocardial infarction and control group each consisted of 187 participants (Table 1). Cases and controls were individually matched by gender, age and place of residence. More than 50% of cases and controls were ≤ 65 years old. The mean age of cases was 63.2 years (standard deviation = 10.5), and the mean age of controls was 63.9 years (standard deviation = 10.4). A total of 72.7% of participants were from urban areas.

**Table 1:** Characteristics of patients with acute myocardial infarction and their controls.

	Cases (n = 187) No. (%)	Controls (n = 187) No. (%)	*P*
**Age (**≤ 65 **years)**	102 (54.5)	96 (51.3)	matched
**Gender (Male**)	113 (60.4)	113 (60.4)	matched
**Place of residence (Urban)**	136 (72.7)	136 (72.7)	matched
Occupation*			
Housewife	30 (16.0)	30 (16.0)	
- Manual worker	95 (50.8)	96 (51.3)	
- Farmer	16 (8.6)	19 (10.2)	
- Clerk	29 (15.5)	23 (12.3)	
- Professional	17 (9.1)	19 (10.2)	0.956
**Educational level (≤ 8 years)**	74 (39.6)	76 (40.6)	0.916
**Marital status (with partner)**	137 (73.3)	133 (71.1)	0.729
**Body mass index (≥ 25kg/m**^**2**^**)**	126 (67.4)	107 (57.2)	0.043
**Oral contraceptive use**	4 (5.6)	7 (9.2)	0.535
**Diabetes mellitus**	39 (20.9)	13 (7.0)	<0.001
**Hypertension**	107 (57.2)	77 (41.2)	0.003
**Hypercholesterolemia**	40 (21.4)	10 (5.3)	<0.001
**Disorders of thyroidea**	13 (7.0)	5 (2.7)	0.088
**Stressful events**	149 (79.7)	116 (62.0)	<0.001
**Family history of myocardial infarction**	109 (58.3)	78 (41.7)	0.002
**Smoking**	119 (63.6)	96 (51.3)	0.021
**Coffee consumption**	165 (88.2)	170 (90.9)	0.499

P, probability value (according to univariate logistic regression analysis).

* For retiree the occupation before retirement was shown.

Cases and controls did not differ significantly in occupation, employment, education level, marital status, oral contraceptive use, coffee consumption. Significantly more participants were overweight (body mass index ≥ 25kg/m^2^) among cases than in controls (*p* = 0.043). Data from personal medical history showed that cases significantly more frequently had diabetes (*p* < 0.001), hypertension (*p* = 0.003) and hypercholesterolemia (*p* < 0.001). Disorders of thyroidea were more frequently recorded in cases than controls (*p* = 0.088. Presence of myocardial infarction in family history was significantly more common in cases (58.3%) than in controls (41.7%), *p* = 0.002. Stressful event was present in 79.7% cases and 62.0% controls (*p* < 0.001). Cases and controls were significantly different in cigarette smoking habits (63.6% cases versus 51.3% controls, *p* = 0.021).

Among participants who had ever consumed alcohol, a higher proportion of cases than controls had body mass index ≥ 25kg/m^2^ (*p* = 0.025), diabetes mellitus (*p* = 0.022), hypertension (*p* = 0.002), hypercholesterolemia (*p* = 0.002), stressful events (*p* = 0.027), and family history of myocardial infarction (*p* = 0.045) (Table 2). Among participants who had never consumed alcohol, a higher proportion of cases than controls had diabetes mellitus (*p* = 0.001), hypercholesterolemia (*p* = 0.001), stressful events (*p* = 0.001), family history of myocardial infarction (*p* = 0.011), and smoking (*p* = 0.041).

**Table 2:** Characteristics of patients with acute myocardial infarction and their controls, according to alcohol use.

	Alcohol use					
	Ever (n = 196)			Never (n = 178)		
	Cases (n = 102) No. (%)	Controls (n = 94) No. (%)	*P*	Cases (n = 85) No. (%)	Controls (n = 93) No. (%)	*P*
**Age (**≤ 65 **years)**	60 (58.8)	58 (61.7)	0.770	42 (49.4)	38 (40.9)	0.292
**Gender (Male**)	89 (87.3)	79 (84.0)	0.546	24 (28.2)	34 (36.6)	0.265
**Place of residence (Urban)**	70 (68.6)	70 (74.5)	0.429	66 (77.6)	66 (71.0)	0.392
Occupation*						
- Housewife	4 (3.9)	6 (6.4)		26 (30.6)	24 (25.8)	
- Manual worker	61 (59.8)	57 (60.6)		34 (40.0)	39 (41.9)	
- Farmer	8 (7.8)	8 (6.4)		8 (9.4)	13 (14.0)	
- Clerk	16 (15.7)	13 (13.8)		13 (15.3)	10 (10.8)	
- Professional	13 (12.7)	12 (12.8)	0.599	4 (4.7)	7 (7.5)	0.497
**Educational level (≤ 8 years)**	25 (24.5)	30 (31.9)	0.269	49 (57.6)	46 (49.5)	0.295
**Marital status (with partner)**	83 (81.4)	71 (75.5)	0.384	54 (63.5)	62 (66.7)	0.753
**Body mass index (≥ 25kg/m**^**2**^**)**	79 (77.5)	59 (62.8)	0.025	47 (55.3)	48 (51.6)	0.624
**Oral contraceptive use**	0 (0.0)	3 (18.8)	0.119	4 (6.7)	4 (6.7)	1.000
**Diabetes mellitus**	17 (16.7)	6 (6.4)	0.022	22 (25.9)	7 (7.5)	0.001
**Hypertension**	53 (52.0)	28 (29.8)	0.002	54 (63.5)	49 (52.7)	0.145
**Hypercholesterolemia**	24 (23.5)	7 (7.4)	0.002	16 (18.8)	3 (3.2)	0.001
**Disorders of thyroidea**	4 (3.9)	1 (1.1)	0.206	9 (10.6)	4 (4.3)	0.108
**Stressful events**	75 (73.5)	55 (58.5)	0.027	74 (87.1)	61 (65.6)	0.001
**Family history of myocardial infarction**	57 (55.9)	39 (41.5)	0.045	52 (61.2)	39 (41.9)	0.011
**Smoking**	74 (72.5)	61 (64.9)	0.249	45 (52.9)	35 (37.6)	0.041
**Coffee consumption**	93 (91.2)	85 (90.4)	0.856	72 (84.7)	85 (91.4)	0.168

P, probability value (according to univariate logistic regression analysis).

* For retiree the occupation before retirement was shown

### Alcohol consumption and myocardial infarction

The history of alcohol consumption in patients with acute myocardial infarction and their controls was not significantly different: the percentage of those that were not consuming alcohol was slightly higher in controls (49.7%) than in cases (45.5%) (Table 3). Our study found that 24.6% of cases and 20.3% of controls first started drinking alcohol before the age of 20 years, and that average duration of consumption for both groups was 30 years or more. All of the patients who regularly used alcohol consumed more than one type of drink. Of all alcoholic beverages, the most commonly used were spirits (especially rakija, a fruit brandy that normally has 40% alcohol content, but home-produced can be typically 50% to 80%, even going as high as 90%) both in cases (44.4%) and controls (42.2%). But, the risk of myocardial infarction associated with types of alcoholic beverages was similar among patients who reported drinking beer, wine, spirits, or mixed beverages. Although average amount of beer (> 1 bottle) consumed per day was more common in cases than in controls, these differences were not statistically significant. Compared to non-drinkers, cases and controls did not differ significantly in the use of certain types of alcoholic beverages. The intensity of alcohol consumption (the total amount of pure alcohol consumed per day) was higher in cases than in controls, but without statistical significance. The habit of drinking at least one drink every day was reported by 14.4% of cases and 11.8% of controls. Compared to non-drinkers, the habit of binge drinking on one occasion at least once a month during the last year preceding the onset of the myocardial infarction for cases and the current illness for controls was significantly more common in cases (25.1%) than in controls (12.8%): adjusted OR = 2.2 (95%CI = 1.2–4.2, *p* = 0.017), *p* for trend = 0.015. No significant differences were observed in the risk of acute myocardial infarction by the history of cessation of alcohol consumption and duration of abstinence.

**Table 3:** Characteristics of study participants according to drinking pattern.

Alcohol consumption history	Cases (n = 187) No. (%)	Controls (n = 187) No. (%)	*OR (95%CI)* Unadjusted	*P*	*OR (95%CI)* Adjusted*	*P*
**Alcohol consumption (Yes)**	102 (54.5)	94 (50.3)	1.2 (0.8–1.8)	0.408	1.2 (0.7–1.9)	0.488
**Time of the beginning of consumption (< 20 years)**	46 (24.6)	38 (20.3)	1.3 (0.8–2.2)	0.290	1.2 (0.6–2.1)	0.616
**Duration of consumption (≥ 30 years)**	66 (35.3)	61 (32.6)	1.2 (0.8–1.9)	0.468	1.2 (0.7–2.0)	0.517
**Alcoholic beverages types**						
Non-drinkers	85 (45.5)	93 (49.7)	1.0^†^		1.0^†^	
Beer	53 (28.3)	39 (20.9)	1.5 (0.9–2.5)	0.125	1.5 (0.8–2.6)	0.191
Wine	50 (26.7)	38 (20.3)	1.4 (0.9–2.4)	0.165	1.4 (0.8–2.5)	0.273
Spirits	83 (44.4)	79 (42.2)	1.2 (0.8–1.8)	0.521	1.2 (0.7–1.9)	0.566
Mixed	102 (54.5)	94 (50.3)	1.2 (0.8–1.8)	0.408	1.2 (0.7–1.9)	0.488
**Average amount of beverages per day**						
Non-drinkers	85 (45.5)	93 (49.7)	1.0^†^		1.0^†^	
Beer (> 1 bottle / day)	12 (6.4)	5 (2.7)	2.6 (0.9–7.8)	0.081	2.7 (0.8–8.6)	0.094
Wine (> 1 glass / day)	9 (4.8)	4 (2.1)	2.5 (0.7–8.3)	0.146	1.6 (0.4–6.3)	0.510
Spirits (> 1 shot / day)	20 (10.7)	16 (8.6)	1.4 (0.7–2.8)	0.394	1.4 (0.6–3.0)	0.429
**The total amount of pure**^‡^ **alcohol (g/day)**						
Non-drinkers	85 (45.5)	93 (49.7)	1.0^†^		1.0^†^	
≤ 13	55 (29.4)	59 (31.6)	1.0 (0.6–1.6)	0.934	2.0 (0.6–1.8)	0.931
> 13	47 (25.1)	35 (18.7)	1.5 (0.9–2.5)	0.153	1.4 (0.8–2.6)	0.231
*p*_trend_				0.326		0.443
**Regular consumers (daily) in the last year**						
Non-drinkers	85 (45.5)	93 (49.7)	1.0^†^		1.0^†^	
Irregular	75 (40.1)	72 (38.5)	1.1 (0.7–1.8)	0.558	1.1 (0.6–1.8)	0.776
Regular (daily)	27 (14.4)	22 (11.8)	1.3 (0.7–2.5)	0.363	1.5 (0.8–3.1)	0.226
*p*_trend_				0.628		0.473
**Binge drinking**^||^						
Non-drinkers	85 (45.2)	93 (49.7)	1.0^†^		1.0^†^	
No binge drinking	55 (29.4)	70 (37.4)	0.9 (0.5–1.4)	0.519	0.9 (0.5–1.4)	0.548
Binge drinking	47 (25.1)	24 (12.8)	2.1 (1-2-3.8)	0.009	2.2 (1.2–4.2)	0.017
*p*_trend_				0.009		0.015
**Cessation of consumption (No)**	82 (43.9)	76 (40.6)	1.2 (0.8–1.8)	0.448	1.2 (0.8–2.1)	0.397
**Duration of abstinence (≤10 years)**	12 (6.4)	7 (3.7)	1.9 (0.7–5.0)	0.207	1.6 (0.5–4.9)	0.398

Abbreviations: OR, Odds Ratio; CI, Confidence Interval; P, Probability value (according to logistic regression analysis); P_trend_, trend in risk (according to logistic regression model).

* Adjusted for body mass index, positive personal medical history for diabetes mellitus, hypertension, hypercholesterolemia, and disorders of thyroidea, family history of myocardial infarction, stressful events, and tobacco use

^†^ Reference category

^‡^ A standard drink is defined as 13 g alcohol

^||^ Binge drinking was defined as the consumption of 5+ standard drinks for men and 4+ standard drinks for women on one occasion at least once a month during the last year preceding the onset of the myocardial infarction for cases and the current illness for controls.

Analysis of binge drinking by age, gender and place of residence revealed that the increase of risk for acute myocardial infarction was associated with older age (adjusted OR = 5.1, 95%CI = 1.7–15.1, *p* for trend = 0.010), male gender (adjusted OR = 2.3, 95%CI = 1.1–5.2, *p* for trend = 0.028) and rural place of residence (adjusted OR = 4.8, 95%CI = 1.3–18.5, *p* for trend = 0.033) (Table 4).

**Table 4:** Estimated risk*† - odds ratio (95% confidence intervals) of acute myocardial infarction in relation to binge drinking in separate strata of selected covariates.

	Binge drinking^‡^			
Covariates	Non-drinkers^||^	No binge drinking	Binge drinking	*p*_trend_
**Age (years)**				
≤ 65	1.0	0.5 (0.2–1.2)	0.9 (0.4–2.2)	0.111
**>** 65	1.0	1.1 (0.5–2.3)	5.1 (1.7–15.1)	0.010
**Gender**				
Male	1.0	0.9 (0.4–2.0)	2.3 (1.1–5.2)	0.028
Female	1.0	0.9 (0.4–2.4)	—	
**Place of residence**				
Rural	1.0	0.9 (0.3–2.7)	4.8 (1.3–18.5)	0.033
Urban	1.0	0.8 (0.4–1.5)	1.3 (0.6–2.8)	0.486

Abbreviation: P_trend_, trend in risk (according to logistic regression model).

* According to multivariate logistic regression analysis

^†^ Adjusted for body mass index, positive personal medical history for diabetes mellitus, hypertension, hypercholesterolemia, and disorders of thyroidea, family history of myocardial infarction, stressful events, and tobacco use

^‡^ Binge drinking was defined as the consumption of 5+ standard drinks for men and 4+ standard drinks for women on one occasion at least once a month during the last year preceding the onset of the myocardial infarction for cases and the current illness for controls

^||^ Reference category.

## Discussion

Our study showed that binge drinking is significantly associated with the increased risk for acute myocardial infarction, especially in older people, males and those living in the countryside.

Until today, a few investigators have specifically examined the influence of binge drinking on myocardial infarction occurrence. Our results (for binge drinking: OR = 2.2, 95%CI = 1.2–4.2) were consistent with some previous studies [27–31] that showed that binge drinking was associated with an increased risk of acute myocardial infarction. Also, a nationwide representative cohort of 50,000 Swedish male conscripts of 18–20 years of age indicated, through a 1969–2004 follow-up, that binge drinking and high alcohol consumption significantly increased the risk of total myocardial infarction (1.71, 95%CI = 1.24–2.35), but non-significantly increased the risk for fatal myocardial infarction (2.23, 95%CI = 0.81–6.53) [35]. Nevertheless, some epidemiological studies have found that heavy alcohol consumption was associated with a decreased risk of coronary heart disease [36,37]. During an average follow up of 4.36 years, Chinese men aged 45–81 years, who consumed >40 grams of ethanol per time with ≥5 times per week, had a significantly decreased risk of coronary heart disease (HR = 0.73, 95%CI = 0.54–1.00) compared with non-drinkers [37]. Contrary to that, a case-crossover study of 250 incident acute myocardial infarction cases in Switzerland found no evidence that the effect of binge drinking was significant [38]. Also, a recent a case-crossover study in Iran showed that alcohol abuse was not an acute trigger associated with significantly increased risk of myocardial infarction [39]. Possible explanations for the differences in results of these studies could include differences both in the characteristics of respondents (in terms of age, gender, occupation, comorbidity) and in the study design (in terms of the differences in selection of the cases with myocardial infarction and their controls, choice of the reference category for assessment of alcohol consumption, etc).

Drinking of alcoholic beverages in Serbia is considered a socially acceptable behavior [40]. The high prevalence of alcohol use (especially spirits) in our study is, inter alia, a consequence of considering alcohol use a part of tradition, customs and culture. Also, 16% of the general population in Serbia practiced excessive drinking per occasion (binge drinking) at least once per month [40]. In our case-control study, binge drinking was reported by 25.1% of cases and 12.8% of controls, which is, according to the available literature data, a larger percentage than in most of the countries in the region [41,42]. In the United States of America, the overall prevalence of binge drinking among adults aged ≥18 years was 17.1% in 2010 [43]. Potential mechanisms for a detrimental effect of binge drinking on acute myocardial infarction risk include increased fluctuations in blood pressure (either an acute increase or sustained hypertension after cessation of drinking) [44,45], together with heightened platelet activation and adverse changes in the balance of fibrinolytic factors, ventricular arrhythmia [46], direct damage to heart muscle cells (alcoholic cardiomyopathy) [47], as well as the unfavorable effects on lipid profile reported in some studies (such as increase of low-density lipoprotein levels, and no increase in high-density lipoprotein levels) [48], although the evidence is inconsistent [49].

The relationship between binge drinking and risk of acute myocardial infarction seems to be associated with older age, male gender and with life in the countryside in our study. Similarly, the “INTERHEART” study recorded that binge drinking related to myocardial infarction, especially in older people in developed countries [26]. Information about potential mechanisms linking diverse patterns of drinking to diverse risk of myocardial infarction by age, gender and place of residence is limited. Heavy episodic drinking is frequently reported by elderly, male and those with lower levels of education, who may be particularly at risk because of age-related increases in comorbidities and medication use [50,51]. Additionally, women had, on average, a very low level of alcohol consumption and there were only small differences in incidence of myocardial infarction between drinkers and non-drinkers [29].

The findings of numerous epidemiological studies that indicated a protective effect of moderate alcohol consumption on the occurrence of myocardial infarction were not confirmed in our study. Tavani et al [18] noted that the consumption of alcoholic drinks was inversely related to non-fatal myocardial infarction, irrespective of the type of drink, and depended on the duration of alcohol consumption. People who consumed alcohol for 40 years or more had a 60% lower risk of developing the disease, and those who regularly consumed alcoholic beverages, one to three glasses a day, had a two times lower risk of myocardial infarction [18]. Schröder et al [15] observed that consumption of up to 30 g of alcohol per day was inversely related to the onset of non-fatal myocardial infarction after controlling for potential confounding factors. Alcohol consumption of up to 20 g / day including wine, beer and brandy, significantly reduced the risk of myocardial infarction, while higher alcohol intake did not substantially reduce the risk [15]. Two nested case-control studies in the United States, which included women enrolled in the Nurses Health Study and men enrolled in the Health Professionals Follow-Up Study, found that the frequency of alcohol consumption was significantly associated with a reduced risk of acute myocardial infarction [52]. The lowest risk was recorded in people who consumed alcohol most frequently (3–7 drinks per week) and those who consumed the highest quantities (30g / day and more). Similar results were observed in studies in Spain, Costa Rica, France and Northern Ireland [53–55]. A large meta-analysis which included 240 studies showed that a daily intake of alcohol in the amount of 50 g / day had a protective effect for myocardial infarction (RR = 0.87, 95% CI = 0.54–0.90), while intake of 100 g / day was associated with an increased risk (RR = 1.13; 95% CI = 1.06–1.21) [56]. This meta-analysis found that the daily intake of 72 g/day was the limit in amount of consumed alcohol for which significant protective effect was still recorded, while the amount of 89 g / day was the value from which a significantly increased risk began [56].

The relationship between alcohol consumption and myocardial infarction remains controversial. Differences between the results obtained in some studies can partly be explained with different methodology that was used, but also with differences in the characteristics of study groups (some studies are limited to specific groups of subjects such as employed patients or patients after rehabilitation, or focused on older patients, while other focus on younger and healthier patients). Although our case-control study was hospital-based, data in available literature show that the frequency of alcohol consumption found in our respondents was similar to the pattern of alcohol consumption across the entire population [40,41]. A possible reason for the absence of substantial differences is the fact that comorbidity was not a reason for exclusion from the study (among the controls there were probably illnesses associated with alcohol consumption, e.g. trauma). Also, in our study among those who used alcohol there were significantly more persons with obesity and hypertension among cases than among controls, while those differences were not significant in non-drinkers. In addition, our study did not include the most severe patients with myocardial infarction, as they died shortly after admission to the hospital, so there was no alcohol consumption data available. Future epidemiological analytical studies are needed to clarify the impact of not only pure ethanol but also non-alcoholic ingredients in alcoholic beverages on the occurrence of myocardial infarction.

### Limitations of the study

Our study has several strengths. Firstly, this study presented detailed qualitative and quantitative data on alcohol consumption over a period of 10 years before the onset of the disease. Secondly, based on the intake of certain types of beverages, we estimated the average daily intake of pure alcohol in our study. In addition, the former drinkers (regardless of the duration of abstinence) were excluded from the reference group which might have minimized bias to some extent. But, our study has some limitations. The limitation of this study might be that the definitions of alcohol consumption (such as differences in the definition of alcohol use, categories of the frequency and intensity of alcohol consumption, as well as the differences in the quality of alcoholic beverages or "standard" drink) somewhat differed from the definitions that were used in some other studies, so the comparison of findings was sometimes difficult [25]. The response rate of 100%, as well as the fact that the interview was always conducted by a medical doctor, could partly reduce the shortcomings of our case-control study. Also, although we estimate that the underreporting of alcohol consumption in this study was not a problem, because drinking in Serbia is a traditionally accepted habit, we cannot preclude the possible existence of recall bias when doing a retrospective study of alcohol use and myocardial infarction. The limitation of the study is the lack of information on the consumption of alcohol in periods of high sensitivity (such as puberty, pregnancy, lactation). In addition to the well-known shortcomings of case control studies, a further limitation of the study was the relatively small sample size. We acknowledge that failure in both determining previous use of medications among cases and controls and compliance to a recommended treatment might have led to a less accurate assessment of risk for myocardial infarction, but in this study there was no suitable data for this. As in many other studies, our list of potential confounders was not complete and we cannot exclude the possibility that some unmeasured confounders (such as dietary factors, socio-economic status) might explain the results of this study.

## Conclusions

Our study is one of the few in which binge drinking was found to be a risk factor for acute myocardial infarction. Binge drinking is frequent among older aged, men and those living in the countryside, and needs to be addressed in understanding the health risks. But, this study confirms that the relationship between alcohol consumption and myocardial infarction is quite complex and further analytical epidemiological studies are needed to clarify the observed association.

## Supporting information

S1 File Question–in Serbian. (DOC)

S2 File Question–in English. (DOC)
