# Supplementary material for: Myocardial infarction and alcohol consumption: A case-control study
Source: PLoS One. 2018 Jun 4;13(6):e0198129. doi: 10.1371/journal.pone.0198129 (PMC5986147; doi:10.1371/journal.pone.0198129)
Supplement: S1 File — (DOC) [file pone.0198129.s001.doc]

**MEDICINSKI FAKULTET U KRAGUJEVCU**

**KLINIČKI CENTAR**

***EPIDEMIOLOŠKI UPITNIK O FAKTORIMA RIZIKA ZA KARDIOVASKULARNE BOLESTI***

Broj ankete: _______ Datum: ________

Broj istorije bolesti ____________

**Ispitanici: kontrola 0 bolesnik 1**

**Telefon: _______________________ Adresa: ________________________**

*I OSNOVNI PODACI*

1. Ime i prezime____________________________ 1a. Pol: 1- muški ; 2- ženski

2. Datum rođenja_____________ 3. Mesto rođenja________________

4. Mesto stalnog boravka_____________________

...

*II SOCIJALNE KARAKTERISTIKE*

11. Zanimanje___________________ 12. Zaposlenost**: Ne Da**

# Za penzionere upisati zanimanje pre odlaska u penziju: ___________________________

13. Koliko ste godina škole ukupno završili_____________

14. Bračno stanje 1. neoženjen-neudata 3. razveden-a

2. oženjen-udata 4. udovac-udovica 5. ponovo oženjen-udata

*...*

*III ANTROPOMETRIJSKE MERE*

25. Koliko ste visoki (cm) _________

26. Kolika je bila Vaša telesna težina u poslednjih godinu dana ( do početka bolesti, odnosno

anketiranja) _________________ BMI: _____________________

*IV ANAMNEZA REPRODUKTIVNOG PERIODA ŽIVOTA*

## A) GINEKOLOŠKA ANAMNEZA

## B) ANAMNEZA TRUDNOĆA I POROĐAJA

## C) KONTROLA RAĐANJA

64. Da li ste do sada koristili pilule za kontracepciju? **Ne Da**

*...*

*V PODACI O IZLOŽENOSTI*

78. Da li se u poslednjih godinu dana pre početka Vaše bolesti ili ranije, dogodila značajnija

promena u životu Vaše porodice (osoba sa kojima živite ili su Vam veoma bliske), bilo kom

članu uključujući i Vas: **Ne Da**

79. Ako DA, navesti: Tokom proteklih godinu dana Pre više od godinu dana

Problemi unutar porodice Da Ne Da Ne

(bračni, finansijski, drugi)

- menopauza Da ___________ ___________ __________ ________

- mobilizacija (vrsta) (srodstvo) (vrsta) (srodstvo)

Da ___________ ___________ __________ ________

(vrsta) (srodstvo) (vrsta) (srodstvo)

Problemi na radnom mestu Da Ne Da Ne

- nezaposlenost Da ___________ ___________ __________ _______

- penzionisanje (vrsta) (srodstvo) (vrsta) (srodstvo)

Da ___________ ___________ __________ ________

(vrsta) (srodstvo) (vrsta) (srodstvo)

Gubici (smrt dece, roditelja, Da Ne Da Ne

supruga) Da ___________ ___________ __________ _______

(vrsta) (srodstvo) (vrsta ) (srodstvo)

Da ___________ ___________ __________ ________

(vrsta) (srodstvo) (vrsta) (srodstvo)

Porodični sukobi Da Ne Da Ne

sa zakonom Da ___________ ___________ __________ _______

(vrsta) (srodstvo) (vrsta) ( srodstvo)

Da ___________ ___________ __________ ________

(vrsta) (srodstvo) (vrsta) (srodstvo)

*VI PODACI O NAVIKAMA*

VI-A- Pušenje

82. Da li pušite ili ste pušili cigarete: 1. ne 2. bivši pušač 3. pušač

(definicija za pušače: ako je redovno pušio do pre mesec dana bar po jednu cigaretu dnevno ili oko 30 gr duvana mesečno tokom jedne godine)

VI-B- Alkohol

93. Da li pijete ili ste pili alkoholna pića: 1- Ne; 2- Da, povremeno ; 3- Da, svakodnevno

94. Ako Da, u koliko godina ste počeli da pijete? ____________________

95. Koliko godina ste pili (pijete)? __________________________

96. Da li ste prestali da pijete? **Ne Da**

97. Koliko dugo ne pijete? _________________________

98. Prosečna količina popijenog pića u vreme najintenzivnijeg konzumiranja?

1-piva (flaša) ____________ 2-vina (čaša) ___________ 3-žestokog pića (čašica) __________

VI-C- Kafa

99. Da li pijete kafu: 1- Ne; 2- Da, povremeno ; 3- Da, svakodnevno

100. Ako DA, koliko prosečno dnevno šoljica konzumirate _________

Koliko dugo ____________

*VII RANIJE BOLESTI I OPERACIJE*

107. Koje ste druge bolesti imali u životu:

Bolest Uzrast Trajanje

1. hipertenzija Ne _____________ __________

2. šećerna bolest Ne ____________ _________

3. hiperholesterolemija Ne ___________ __________

4. oboljenje štitne žlezde Ne ____________ _________

*VIII PORODIČNA ANAMNEZA*

109. Navedite da li je bilo srodnika koji su bolovali ili umrli od infarkta miokarda? **Ne Da**

| NAMIRNICA  *Pića i napici* | šifra | br.  m. | učestalost konzumiranja D,N,M,G | jedinica | br.j. |  |
| --- | --- | --- | --- | --- | --- | --- |
| … |  |  |  |  |  |  |
| Rakija | 008 |  | , | 0.5 dl |  |  |
| Vinjak | 009 |  | , | 0.5 dl |  |  |
| Konjak | 010 |  | , | 0.5 dl |  |  |
| Votka | 011 |  | , | 0.5 dl |  |  |
| Viski | 012 |  | , | 0.5 dl |  |  |
| Liker | 013 |  | , | 0.5 dl |  |  |
| Vermut | 014 |  | , | 1.0 dl |  |  |
| Vino belo | 015 |  | , | 2.0 dl |  |  |
| Vino roze | 016 |  | , | 2.0 dl |  |  |
| Vino crno | 017 |  | , | 2.0 dl |  |  |
| Pivo | 018 |  | , | 2.0 dl |  |  |

**….**
