# Supplementary material for: Myocardial infarction and alcohol consumption: A case-control study
Source: PLoS One. 2018 Jun 4;13(6):e0198129. doi: 10.1371/journal.pone.0198129 (PMC5986147; doi:10.1371/journal.pone.0198129)
Supplement: S2 File — (DOC) [file pone.0198129.s002.doc]

**MEDICAL FACULTY KRAGUJEVAC**

**CLINICAL CENTER**

***EPIDEMIOLOGICAL QUESTIONNAIRE FOR RISK FACTORS FOR CARDIOVASCULAR DISEASES***

Questionnaire number: _______ Date: ________

Patient's history number ____________

**Participants: control 0 case 1**

**Phone number: ____________________ Address: ________________________**

*I GENERAL INFORMATION*

1. First and last name____________________________ 1a. Sex: 1- male ; 2- female

2. Date of birth_____________ 3. Place of birth________________

4. Place of residency_____________________

...

*II SOCIAL CHARACTERISTICS*

11. Occupation___________________ 12. Employment**: No Yes**

# For retiree enter occupation before retirement: ___________________________

13. Educational level (years in school)_____________

14. Marital status 1. single 3. divorced

2. married 4. widowed 5. remarried

*...*

*III ANTHROPOMETRIC MEASUREMENTS*

25. How tall are you (cm) _________

26. What has your weight been in the last year (prior to the beginning of your disease, or prior to this survey) _________________ BMI: _____________________

*IV REPRODUCTIVE ANAMNESIS*

## A) GYNECOLOGICAL HISTORY

## B) HISTORY OF PREGNANCIES AND CHILDBIRTH

## C) BIRTH CONTROL

64. Have you ever used birth control pills? **No Yes**

*...*

*V INFORMATION ABOUT EXPOSURE*

78. Was there any significant change in your family (persons you live with or are close to), any member including you in the last year prior to the beginning of your disease or prior to this survey: **No Yes**

79. If YES, state: During the last year Over one year ago

Problems inside the family Yes Ne Yes Ne

(marital, financial, other)

- menopause Yes ___________ ___________ __________ ________

- mobilization (type) (relation) (type) (relation)

Yes ___________ ___________ __________ ________

(type) (relation) (type) (relation)

Problems at work Yes No Yes No

- unemployment Yes ___________ ___________ __________ _______

- retirement (type) (relation) (type) (relation)

Yes ___________ ___________ __________ ________

(type) (relation) (type) (relation)

Losses (death of children, parents, Yes No Yes No

marital partners) Yes ___________ ___________ __________ _______

(type) (relation) (type ) (relation)

Yes ___________ ___________ __________ ________

(type) (relation) (type) (relation)

Family issues with Yes No Yes No

the law Yes ___________ ___________ __________ _______

(type) (relation) (type) ( relation)

Yes ___________ ___________ __________ ________

(type) (relation) (type) (relation)

*VI INFORMATION ABOUT HABITS*

VI-A- Smoking

82. Do you smoke or have smoked cigarettes: 1. no 2. ex smoker 3. smoker

(The participant was a smoker if he regularly smoked at least one cigarette per day or approximately 30 g of tobacco per month for one year.)

VI-B- Alcohol

93. Do you drink or have drunk alcohol: 1- no; 2- yes, occasionally; 3- yes, everyday

94. If YES, at what age did you start drinking? ____________________

95. For how many years have you drunk (or still do)? __________________________

96. Have you stopped drinking? **No Yes**

97. How long are you abstinating? _________________________

98. What is the average amount of alcohol you had at the time of the most intense consumption?

1-beer (bottle) ____________ 2-vina (glass) ___________ 3-spirit (shot) __________

VI-C- Coffee

99. Do you drink coffee: 1- No; 2- yes, occasionally ; 3- yes, every day

100. If YES, how many cups a day do you drink _________

For how long ____________

*VII PREVIOUS DISEASES AND SURGERIES*

107. Which other diseases did you have:

Disease Onset Duration

1. hypertension No _____________ __________

2. diabetes mellitus No ____________ _________

3. hypercholesterolemia No ___________ __________

4. disorders of thyroidea No ____________ _________

*VIII FAMILY HISTORY*

109. Do you have any relatives that suffered or died from myocardial infarction? **No Yes**

| FOOD  *Drinks and beverages* | code | No.  m. | consumption frequency D,W,M,Y | unit | No.u. |  |
| --- | --- | --- | --- | --- | --- | --- |
| … |  |  |  |  |  |  |
| Rakia | 008 |  | , | 0.5 dl |  |  |
| Cognac | 009 |  | , | 0.5 dl |  |  |
| Brandy | 010 |  | , | 0.5 dl |  |  |
| Vodka | 011 |  | , | 0.5 dl |  |  |
| Whiskey | 012 |  | , | 0.5 dl |  |  |
| Liqueur | 013 |  | , | 0.5 dl |  |  |
| Vermouth | 014 |  | , | 1.0 dl |  |  |
| White wine | 015 |  | , | 2.0 dl |  |  |
| Rose wine | 016 |  | , | 2.0 dl |  |  |
| Red wine | 017 |  | , | 2.0 dl |  |  |
| Beer | 018 |  | , | 2.0 dl |  |  |

**….**
